# Functional MRI for Treatment Evaluation in Patients with Head and Neck Squamous Cell Carcinoma: A Review of the Literature from a Radiologist Perspective

**DOI:** 10.1007/s40134-018-0262-z

**Published:** 2018-01-22

**Authors:** Roland P. Nooij, Jan J. Hof, Peter Jan van Laar, Anouk van der Hoorn

**Affiliations:** 1 Department of Radiology, Medical Spectrum Twente, Enschede, The Netherlands; 2 Department of Radiology, University Medical Center Groningen, University of Groningen, Hanzeplein 1, P. O. Box 30.001, 9700 RB Groningen, The Netherlands; 3 Medical Imaging Center, University Medical Center Groningen, University of Groningen, Groningen, The Netherlands

**Keywords:** MRI, Treatment evaluation, Primary tumor, Lymph nodes, Head/neck squamous cell carcinoma, Review

## Abstract

**Purpose of review:**

To show the role of functional MRI in patients treated for head and neck squamous cell carcinoma.

**Recent findings:**

MRI is commonly used for treatment evaluation in patients with head and neck tumors. However, anatomical MRI has its limits in differentiating between post-treatment effects and tumor recurrence. Recent studies showed promising results of functional MRI for response evaluation.

**Summary:**

This review analyzes possibilities and limitations of functional MRI sequences separately to obtain insight in the post-therapy setting. Diffusion, perfusion and spectroscopy show promise, especially when utilized complimentary to each other. These functional MRI sequences aid in the early detection which might improve survival by increasing effectiveness of salvage therapy. Future multicenter longitudinal prospective studies are needed to provide standardized guidelines for the use of functional MRI in daily clinical practice.

## Introduction

Head and neck cancer affects 550,000 new cases and 380,000 deaths worldwide annually [1–3]. Head and neck squamous cell carcinomas comprise over 90% of the head and neck carcinomas [4]. Patients frequently present with a locally advanced stage for which the current therapy is multimodal including surgery, radiation therapy and/or chemotherapy [5–8]. Many patients demonstrate unfavorable treatment response, with locoregional recurrence seen in about 30–60% [7]. This is in about 2/3 due to primary tumor recurrence, 1/3 due to regional nodal metastasis and in 1/3 due to both primary tumor recurrence as well as regional nodal metastasis [9].

Conventional anatomical MRI techniques are commonly used for treatment evaluation, but are often not able to have reliable assess treatment response. Surgery as well as chemoradiotherapy induce false positives on imaging as a result of inducing benign changes involving architectural distortion, fibrosis and/or necrosis [10••, 11, 12, 13•, 14•]. These benign treatment-induced changes should be differentiated from residual and/or recurrent tumor on imaging to prevent unjustified alteration in treatment plan, e.g. salvage therapy or (dis)continuation of therapy. Early detection of local recurrence could lead to timely salvage therapy which can lead to an increase in overall survival [15–17].

Post-treatment surveillance can consist of ultrasound, PET-CT [18–21] and MRI [10••, 11, 12, 13•, 14•]. Several recent studies have shown the potential usefulness of functional MRI techniques for treatment evaluation in patients with head and neck tumors [10••, 11, 12, 13•, 14•, 22••, 23–28, 29•, 30–33•]. Diffusion-weighted imaging is used to image changes in cytoarchitecture and measure cellular density. Perfusion-weighted MRI techniques can identify tumor-induced neovascularization. Changes in concentrations of metabolites are shown with magnetic resonance spectroscopy (MRS).

This review will analyze the functional MR imaging sequences with regards to their possibilities and limitations in head and neck squamous cell carcinoma. Clinical implications, applicability and possibilities of these sequences for treatment evaluation will be addressed.

## Role of Conventional Anatomical MRI in Head and Neck SCC

Conventional anatomical MRI techniques are used for treatment evaluation. MRI is superior to CT yielding higher anatomical detail [11, 12, 13•, 14•, 34–37]. Anatomical MRI to assess HNSCC should include a T1 without fat suppression, T2 with and without fat suppression and T1 post-contrast with fat suppression. These sequences are used to analyze certain characteristics of the primary tumor and possible nodal involvement [11, 12, 13•, 14•, 34–37].

However, anatomical MRI techniques are often unable to accurately identify treatment response showing a pooled sensitivity and specificity for local treatment response evaluation in HNSCC of 84 and 82%, respectively [22••]. This is due to benign treatment effects such as inflammation, fibrosis and necrosis as a result of surgery and chemoradiotherapy. These post-therapy changes show overlapping signal characteristics with tumor. Most problematic for the primary site is that inflammation and tumor both show high T2 signal and enhancement after contrast injection. Lymph node assessment is most hindered by reactive lymph node that can be slightly enlarged similar to nodal metastasis. Furthermore, normal sized nodes can still contain tumor. See Table 1 for a detailed description of the signal intensities on anatomical MRI post-treatment.

**Table 1:** Use of conventional anatomical MRI for treatment evaluation

Anatomical MRI sequence	Primary tumor	Lymph nodes
T1 without fat suppression	Anatomical details Tumor: ↓ compared to fat Fat infiltration by tumor or inflammation: similar ↓/↓↓ Necrosis: ↓↓ round, oval, well circumscribed Fibrosis: Linear commonly ↓↓, but can be ↓/= as well	Anatomical localization of node levels Metastatic lymph nodes: Size ↑/↑↑ (suggested cut-off > 7–10 mm for level II and > 5–7 mm for all other levels). Round shape Reactive lymph nodes: Size =/↑ (can be false-positive using above cut-off); Oval with fatty hilum Location of lymph node and level in relation to location primary tumor
T2 with and without fat suppression	Fat suppression useful for the detection of abnormalities T2 without fat suppression for anatomical details Edema, fat infiltration by tumor or inflammation: similar ↑/↑↑ Necrosis: ↑↑ round, oval, well circumscribed Perineural spread: ↑ Fibrosis: Linear commonly ↓↓, but can be ↓/= as well.	Fat suppression needed to identify abnormal nodes T2 without fat suppression for anatomical details Metastatic lymph nodes: ↑ slightly heterogeneous; more commonly an irregular border; possible extra-nodal extension Reactive lymph nodes: =
T1 post-contrast with fat suppression	Fat infiltration by tumor or inflammation: similar ↑/↑↑ Edema or necrosis: no enhancement Fibrosis: no enhancement after 6-12 months. Most commonly ↑/↑↑ Perineural spread: ↑↑	Fat suppression needed to identify abnormal lymph nodes Metastatic lymph nodes: ↑/↑↑, thick, irregular rim enhancement in case of necrosis Reactive lymph nodes: =/↑

High signal intensity is indicated as ↑, low signal intensity is indicated as ↓ and intermediate signal as =

Higher diagnostic accuracy than 84% sensitivity and 82% specificity post-therapeutically is needed to differentiate treatment effects from true malignancy for the local tumor site and the regional lymph nodes to reliable either initiate new therapy, adjust the current therapy or discontinue unjustified therapy.

## Technical Background of Functional MRI Techniques

### Diffusion Weighted Imaging

DWI measures cellular density and cytoarchitecture using the measurement of water diffusivity. Random diffusion results from the Brownian motion of water molecules. Motion of water molecules is hindered, restricted, by interactions with other molecules and cellular barriers such as fibers, cell membranes and macromolecules. Diffusion abnormalities of water molecules thus reflect changes of tissue organization at a cellular level affecting the MR signal of a DWI sequence as can be seen in a number of processes including malignancy [10••, 11, 12, 13•, 14, 22••, 24••, 25••, 26–28, 29•, 30, 32, 33•].

DWI sequences are based on a T2-weighted sequence. At least two *b* values are needed to analyze motion of water. DWI is done at different *b* values (in s/mm^2^), which represent the duration between the gradient pulses used. Simplified, it is the time that water is allowed to diffuse before the distance is measured. Most commonly, a *b*0 and *b*800 or *b10*00 value are used for head and neck imaging. Diffusion is quantified using ADC in mm^2^/s. Having measured at least two different *b* values (e.g. *b*0 and *b*800), the logarithm of relative signal intensity of a tissue is plotted on the *y* axis against the *b* values on the *x* axis. The slope of the line fitted through the plots describes the ADC. This mono-exponential fitting represents a rough approximation of ADC and is most often used in clinical routine. This parameter is independent of the magnetic field strength. Lower values indicate more restricted diffusion. However, mono-exponential fitted ADC values cannot separate the pure molecular diffusion from the motion of water molecules in the capillary network [32]. Low *b* values are most influenced by the capillary component which influences the ADC values. Multi-exponential models using several *b* values are more suitable for accurate quantification of diffusion without perfusion contamination [30, 32, 33•].

Acquiring multiple *b* values yields techniques such as intravoxel incoherent motion (IVIM) and diffusion kurtosis imaging (DKI). IVIM imaging can distinguish between pure molecular diffusion and motion of water molecules in the capillary network through a single DWI acquisition technique if both low *b* values (< 200 s/mm^2^) and high *b* values (> 200 s/mm^2^) are used. The relationship between signal intensities and multiple *b* values can be assessed. Real diffusion of water molecules (D) can be distinguished from the contribution of perfusion to the signal decay (D*) and the contribution of perfusion to the diffusion signal (f). Another, multiple *b* value method, DKI, represents the extent to which the diffusion pattern of the water molecules deviates from a perfect Gaussian curve that is assumed calculating standard ADC values. Table 2 includes the most commonly used parameters for the different diffusion techniques.

**Table 2:** Use of functional MRI for treatment evaluation

Functional MRI sequence	Most used parameters	During treatment primary tumor and lymph nodes	After treatment primary tumor	After treatment lymph nodes
Diffusion	DWI: ADC, ADC-ratio (= ADC_2000_/ADC_1000_ × 100%) IVIM: D, D*, *f* DKI: skewness of distribution	Locoregional control: %ADC ↑ tumor and lymph nodes Locoregional failure: %ADC ↓ tumor and lymph nodes. Cut-off range 14–24% [32, 55, 65]	Tumor: ADC↓↓ and *b*800-1000 ↑↑ Peritumoral inflammation: ADC ↓/= and *b*800–1000 =/↑ Necrosis/apoptosis: ADC ↑/↑↑ and *b*800–1000 ↑/↑↑ Edema: ADC =/↑ and *b*800–1000 =/↑ Fibrosis: ADC = and *b*800–1000 = IVIM/DKI: ?	Metastatic lymph nodes: ADC ↓↓ and *b*800–1000 ↑↑. Suggested ADC cut-off 1.1 × 10^−3^ mm^2^/s Reactive lymph nodes: ADC ↓/= and *b*800–1000 =/↑ IVIM-derived D and f contradicting literature [35, 38] DKI: ?
Perfusion	DCE: AUC, Ktrans, rate constant, extravascular volume and plasma space volume or flow DSC: blood volume, blood flow, mean transit time, wash out ASL: blood flow	Local control: Ktrans =/↑ [42]. AUC =/↑ [42]. Plasma flow =/↑ [66]. Local failure: Ktrans ↓/= [42] AUC ↓/= [42] Plasma flow ↓/= [66] Regional control (lymph nodes): ?	Tumor: Ktrans ↑, blood volume ↑, blood flow ↑, wash out ↑ Peritumoral inflammation: =/↑ Necrosis/apoptosis: all ↓ Edema: ↓/=/↑ ? Fibrosis: all ↓	Metastatic lymph nodes: blood flow ↑, blood volume ↑, Ktrans ? Reactive lymph nodes: blood flow =/↑, blood volume =/↑, Ktrans ?
Spectroscopy	Concentration of lactate (1.3 ppm), *N*-acetyl-aspartate (2.0 ppm), creatine (3.0 ppm) and choline (3.2 ppm). Ratios can be calculated	Increased choline, decreased creatine and increase choline/creatine ratio in primary tumor recurrence and nodal metastasis is suggested, although insufficient data available to reliably provide insight [62–64]		

See technique section of the paper for explanation of the most commonly used parameters. Suggested cut-off values are given if available. High values are indicated as ↑, low values are indicated as ↓ and intermediate values are indicated as = . References are given if relevant with numbers corresponding to the reference listed in the text

*ADC* apparent diffusion coefficient, *ASL* arterial spin labeling, *AUC* area under the curve, *IVIM* intravoxel incoherent motion, *D* diffusion of water molecules, D* perfusion contribution to the signal decay, *DCE* dynamic contrast enhanced, *DKI* diffusion kurtosis imaging, *DSC* dynamic susceptibility enhanced, *f* contribution of perfusion to the diffusion signal, *Ktrans* capillary permeability, *ppm* parts per million

### MR Perfusion

Perfusion is defined as the steady-state delivery of blood to tissue. Several perfusion techniques are available; dynamic contrast-enhanced (DCE) perfusion, dynamic susceptibility contrast (DSC) perfusion and arterial spin labeling (ASL) all yielding different parameters (see Table 2).

DCE perfusion is most commonly used for the head and neck area. DCE is based on the T1 relaxivity effects of contrast agents. DCE perfusion has been reported as a technique which is able to characterize perfusion and vascularization of tissues [24••, 25••, 30–33•]. However, this has not always been histologically confirmed [25••, 38, 39]. Ktrans is the most commonly derived quantitative parameters representing capillary permeability and seems to be to most consistent parameter [24••, 25••, 40].

DSC perfusion exploits the susceptibility-induced signal loss after administration of contrast on T2-weighted sequences, most commonly a quick T2* gradient echo sequence. It is based on inhomogeneity of the magnetic field during the passage of a short bolus of contrast through a capillary bed [27]. As result on the T2* sequence, blood products, calcifications and aerated structures result in artificial signal loss. Mean transit time, blood flow and blood volume can be calculated. However, in the head and neck area a multitude of artifacts are present (e.g. voluntary/involuntary motion, breathing, air-to-tissue surface artifacts) [10••, 11, 12, 13•, 14•], affecting the reliability of the results acquired with DSC.

ASL is a perfusion technique without injection of contrast. Arterial blood is magnetized below the volume of interest. After a certain period, the magnetized blood flows into the volume of interest and its derived signal is measured. Blood flow can be calculated, which could reflect neovascularity and angiogenic activity of malignancy [32]. ASL also uses T1 relaxation, but is challenging as timing of the signal read-out should be precise. Acquiring the volume of interest too late, and the magnetized arterial blood has already passed. However, ASL is feasible in head and neck cancer using an Locker–Locker sequence [41] or a pseudo-continuous sequence [42].

### MR Spectroscopy

MRS is a technique that detects the presence of specific metabolites. Different metabolites have small differences in their intrinsic vibration frequency and thereby result in small differences in signal of ^1^H protons. Spectroscopy is thus well-suited to detect changes in the components of tissue due to tumor after suppression of the abundant water signal [43]. Single voxel and multivoxel techniques are able to characterize tissue including the measurements of lactate, *N*-acetylaspartate, creatine and choline. Spectroscopy should be regarded as complimentary to the already acknowledged functional MRI techniques in assessing HNSCC.

## Response Evaluation During Therapy

### Diffusion Weighted Imaging

**Fig. 1:**
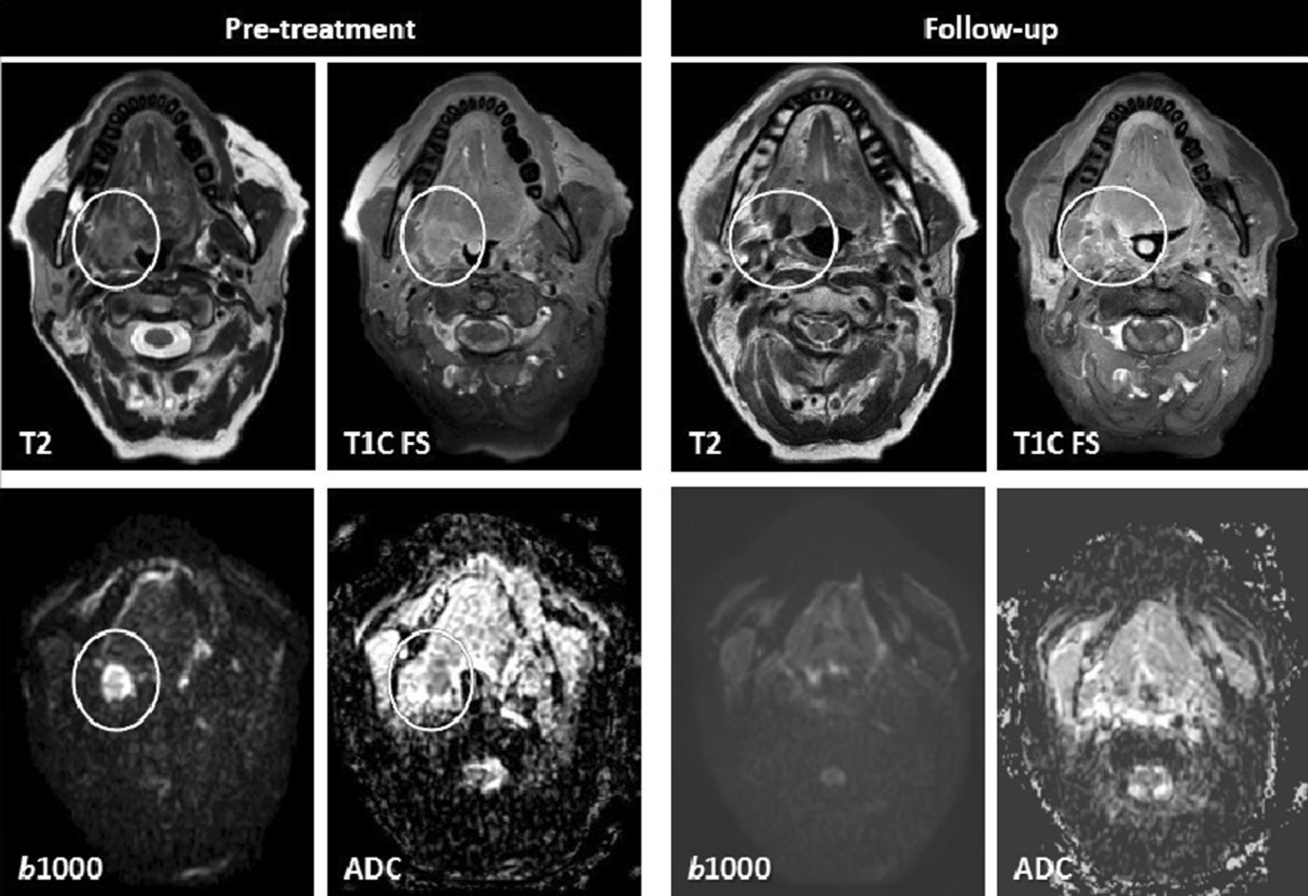
Tumor response confirmed on diffusion. A 54-year-old patient with a tumor at the retromolar trigonum showing high T2 signal, enhancement and diffusion restriction before treatment. Follow-up 6 months after radiation therapy showed at least partial response on anatomical MRI with some residual high T2 signal and enhancement. Diffusion restriction aided in the differentiation between residual tumor and post-therapy inflammation. Lack of diffusion restriction in this patient was in keeping with post-therapy changes

A rise in ADC is seen after the treatment in HNSCC (Fig. 1) and can be seen already in the first few weeks [24••, 29•]. This percentage increase in ADC has been shown to be a predictor of treatment response [24••]. A smaller mean ADC in the first 3 weeks after treatment start was shown in patients with disease failure compared to those with disease control [29•, 33•, 44]. Three other studies found thresholds of < 14–24% to be predictive for regional failure in using clinical outcome data with at least 2 years follow-up [26, 45, 46]. However, it must be noted that imaging is generally not performed within the first couple of weeks in standard clinical practice.

It is of great importance to interpret ADC analysis in conjunction with anatomical imaging. Areas of necrosis may take longer to resolve than solid areas. In the interim, the necrosis may become organized and show a fall in ADC value [24••]. Therefore, it is critical to identify sites of necrosis that need to be excluded from ADC analysis [24••]. Furthermore, the development of mature scar tissue may also decrease the ADC value [27]. The same holds for compact fibrosis which can demonstrate lowered ADC values and low to intermediate T2 signal.

### MR Perfusion

Vascular HNSCCs are thought to have better treatment response compared to less vascular HNSCCs because of better delivery of chemotherapeutic agents and greater radiosensitivity [24••]. On the other hand, vascular tumors may have a poorer outcome because they are thought they have greater metastatic potential [24••]. Reports suggest that a fall in blood volume is associated with poor overall survival. On the other hand, an increased area under the curve is associated with local control [39]. The early rise in volume transfer (Ktrans) is speculated to result from damaged blood vessels causing them to temporarily become leakier, which potentially could increase the delivery of chemotherapeutic agents into the tumor.

Also plasma flow has shown to react in patients undergoing induction chemotherapy for the regional tumor [47]. The median baseline tumor plasma flow was 53 ml/100 ml/min in 25 responders and 24 ml/100 ml/min in 12 non-responders. In lymph nodes, differences were not significantly different between non-responders and responders [47]. After appropriate validation, this method may be potentially used to guide treatment modification in patients.

### MR Spectroscopy

To the best of our knowledge, only one in vitro study of tumor specimens by has shown significantly elevated pre-treatment choline-to-creatine ratios in a poor response group, but these findings could not be confirmed in an in vivo human study using choline/creatine ratios as well choline/water ratios [48].

## Imaging Primary Tumor Site Post-therapy

### Diffusion Weighted Imaging

**Fig. 2:**
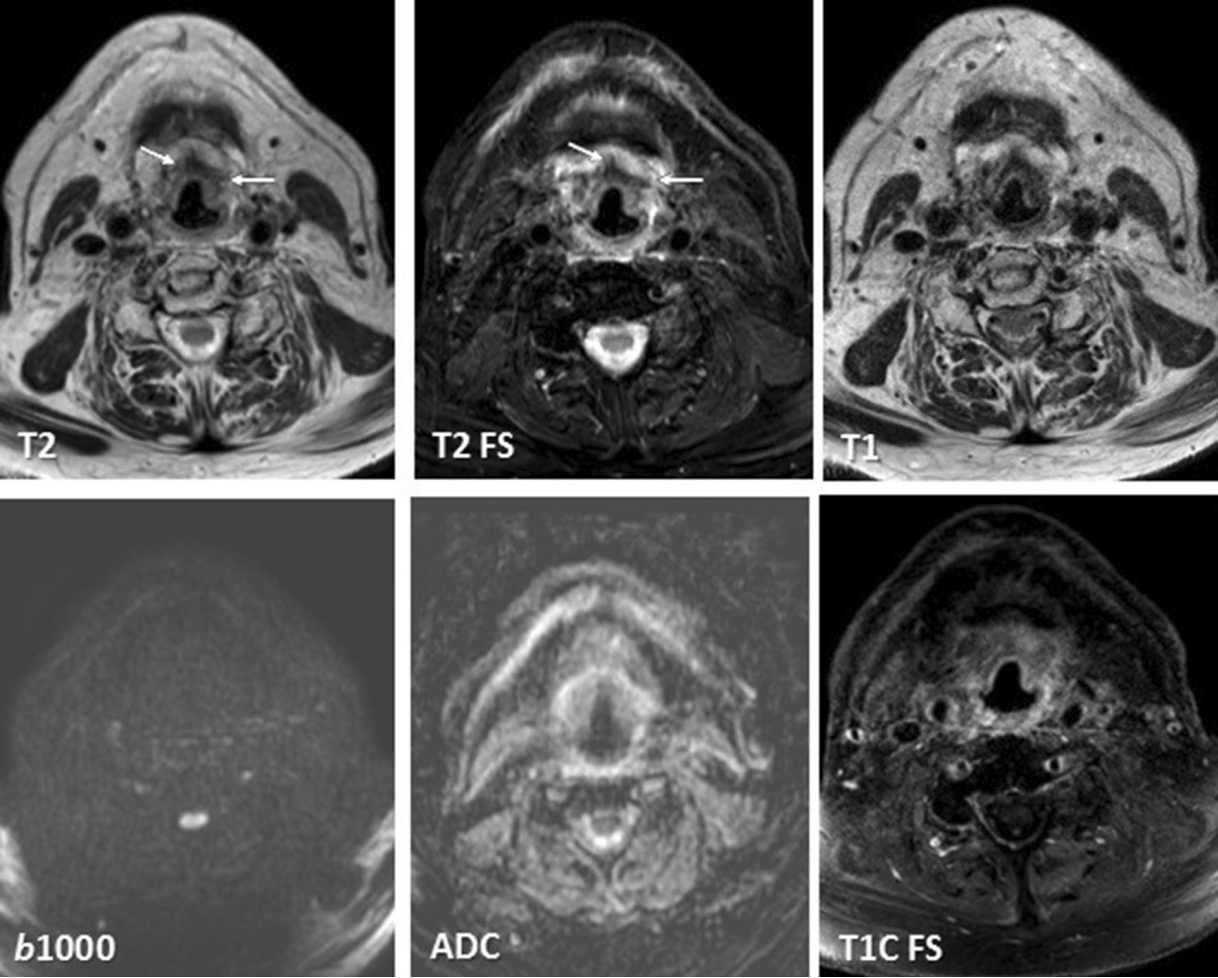
Fibrosis on follow-up MRI confirmed with diffusion. A 67-year-old patient with a T3 vallecula tumor showed fibrosis after radiation therapy with low signal on T1 and T2, no enhancement and no diffusion restriction

Anatomical MRI is mandatory for an accurate delineation of anatomical details (see Table 1). However, anatomical MRI is hindered by interpretation difficulties in the detection of local primary tumor recurrence [10••, 11, 12, 13•, 14•]. A diffusion-derived *b* 800 or *b*1000 map provides high lesion-to-background contrast, outperforming conventional T2-weighted sequences in this aspect. The accompanying ADC indicates whether the high signal on the *b* value map is indeed due to tumor recurrence if low signal is seen on the ADC map. If the high signal on the *b* value map is accompanied by high signal on the ADC map it is not due to tumor and represents T2-shine-through, or increased diffusivity (see also Table 2 for interpretation of functional MRI). Fibrosis also lacks diffusion restriction (Fig. 2). A large meta-analysis showed a higher diagnostic accuracy for ADC compared to anatomical MRI. Anatomical MRI yielded a pooled sensitivity and specificity of 84 and 82%, respectively. ADC showed a pooled sensitivity and specificity of 89 and 86%, respectively [22••]. More recent studies demonstrate a similar diagnostic accuracy for ADC values [46]. Even higher *b* values up to *b*2000 do not increase the diagnostic accuracy [44, 49]. Using both a *b*1000 and *b*2000 and ADC_ratio_ (= ADC_2000_/ADC_1000_ × 100%) can be calculated. The ADC_ratio_ might increase the diagnostic accuracy although results are variable with a sensitivity and specificity of 63 and 84%, respectively, for one study [44]. This is a small study with 32 patients, thus should be further studied in a large population.

Diffusion restriction results from high cellularity as in tumor, but can be also induced due to inflammation and abscesses. Moreover, restricted diffusivity can be seen in normal structures (e.g. Waldeyer’s ring or normal lymph nodes) because these structures have an inherent high cellularity [10••, 11, 12, 13•, 14•]. Apoptosis and tumor necrosis can lead to decreased cellularity resulting in an increased diffusivity [24••, 25••, 29•]. This should be kept in mind when interpreting DWI.

### MR Perfusion

**Fig. 3:**
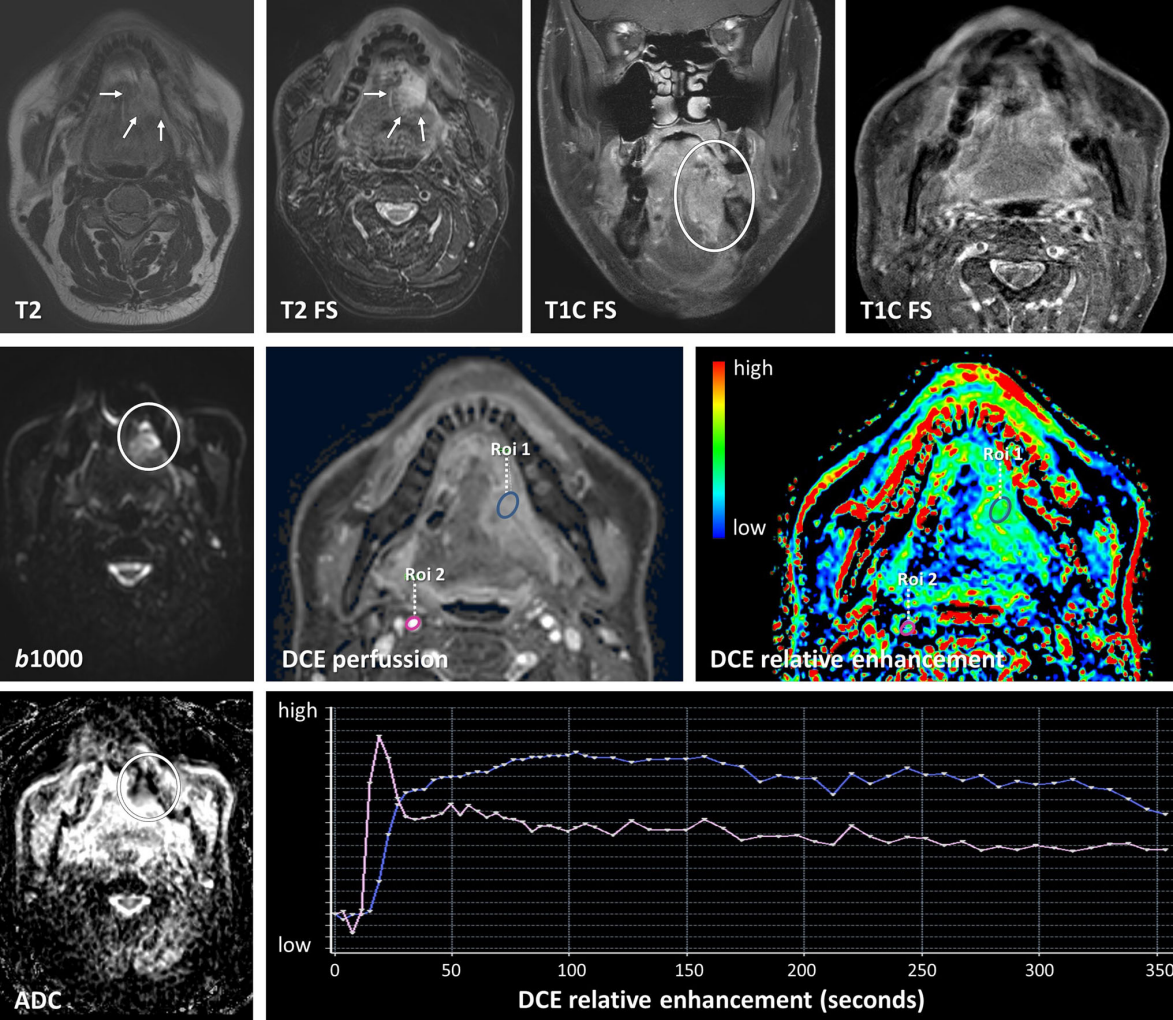
Tumor recurrence differentiated using diffusion and perfusion. A 57-year-old patient with a total resection of a pT2N0Mx lateral tongue carcinoma. Because of small free resection margins, a second resection was performed 1 month later with a submandibulectomy and free radial forearm flap reconstruction. Anatomical MRI showed changes during follow-up 6 months after resection with high signal on T2 with and without fat suppression. There is enhancement post gadolinium. Anatomical MRI was difficult to interpret as these findings could be due to both tumor recurrence as well as inflammation. Functional MRI demonstrated findings in keeping with tumor recurrence. Diffusion restriction was shown with high *b*1000 and low ADC values. Perfusion demonstrated increased AUC. Relative enhancement of the tumor (blue) showed a wash-in comparable to the carotid artery (purple) with plateau phase indicative for tumor. Tumor recurrence was pathologically confirmed (Color figure online)

**Fig. 4:**
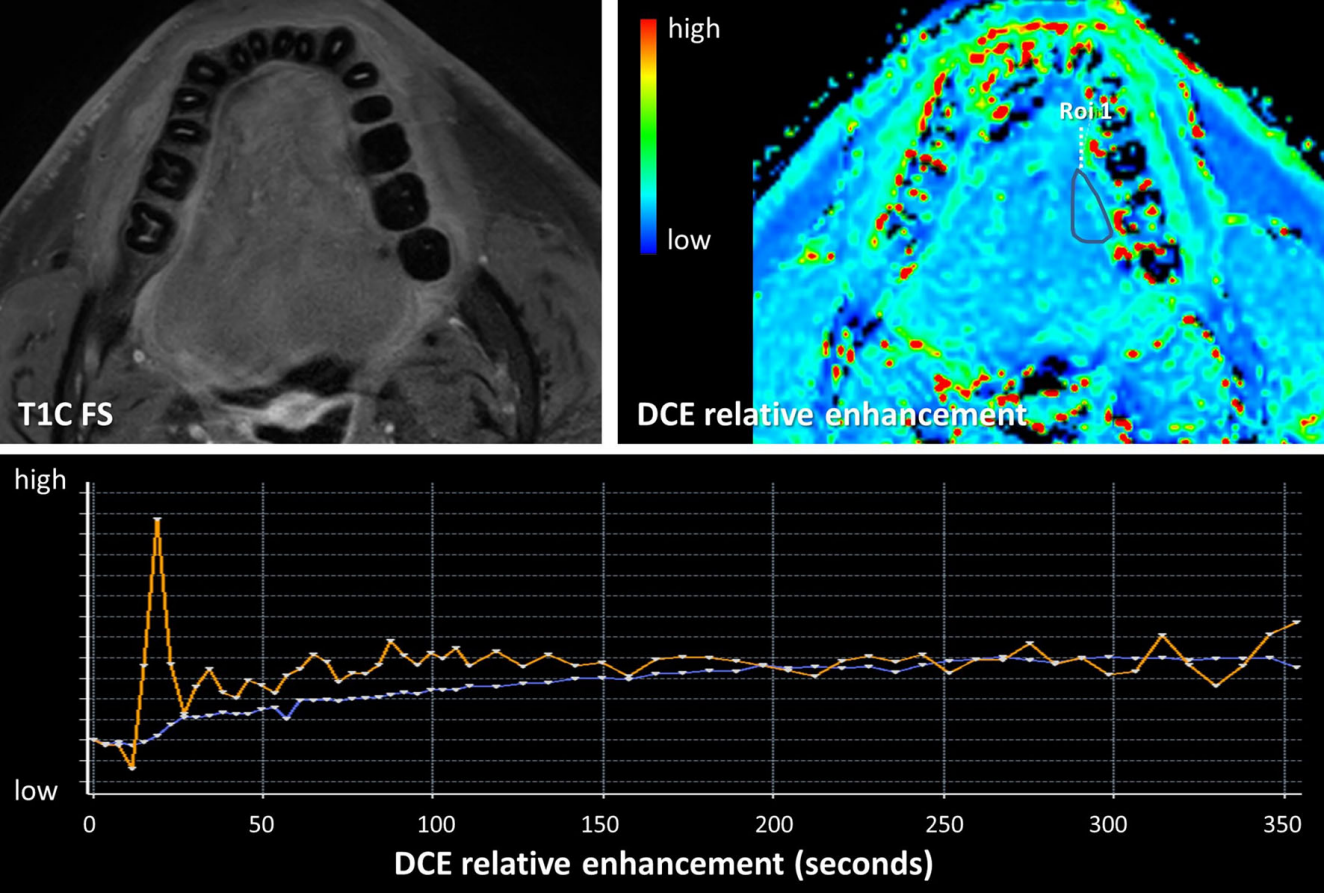
Benign perfusion profile post-therapy. A 45-year-old patient with a T1 tongue carcinoma after resection. The primary site showed some enhancement after gadolinium injection on the T1 with fat suppression. A benign perfusion profile is seen with slowly progressive relative enhancement (Color figure online)

A cross-sectional study demonstrated significant differences between DCE perfusion parameters comparing the blood volume of scar tissue and tumor recurrence in HNSCC [50]. Its potential use in treatment follow-up was also shown in a small retrospective study [51]. Although DSC is not the most used perfusion method in the head and neck area, a higher wash-in on DSC has been related with tumor recurrence instead of treatment changes in a prospective study [33•]. However, diagnostic accuracy studies to differentiate treatment changes from tumor recurrence or residual with DCE or DSC perfusion are lacking. Although, visual assessment is possible (see also Table 2 for interpretation of functional MRI), further quantification is currently hindered by standardization of scan parameters and thresholds. In our experience, the area under the curve (AUC) summing the enhancement in a certain voxel, delineates abnormalities most easily with high values for tumor. Relative enhancement provides more insight in the magnitude of enhancement compared with the pre-contrast values. Region of interest analyses could demonstrate relative enhancements curves with the internal carotid artery as reference. A rapid wash-in comparable with the carotid artery followed by a wash out or plateau phase is indicative of tumor (Fig. 3), while slowly progressive enhancements indicate benign treatment changes (Fig. 4).

### MR Spectroscopy

MRS is not routinely used for the treatment evaluation of HNSCC. However, the presence of choline as indication of proliferation and cell membrane turnover yield high specificity of 100%, although false-negative are frequently present, resulting in a very low sensitivity of 44% [52].

## Imaging Lymph Nodes Post-therapy

### Diffusion Weighted Imaging

Treatment evaluation of regional lymph node is less studied than the primary tumor site. A higher diagnostic accuracy for ADC over anatomical MRI is suggested [22••, 53–55]. Anatomical MRI sensitivity and specificity ranged between 67–90 and 33–97%, respectively [22••]. For ADC, this was 78 and 88% in one study and 73 and 100% in another study [45, 53]. However, the difference was statistically not significant. Benign lymph nodes demonstrate higher ADC values compared to malignant lymph nodes [54–56]. This is also demonstrated in lymph nodes between 5 and 10 mm [54–56]. However, mean ADC values for benign lymph nodes range from to 1.1 to 1.6 × 10^−3^ mm^2^/s, while HNSCC metastatic nodes range between to 0.78 and 1.1 × 10^−3^ mm^2^/s [24••, 25••]. A threshold of 1.1, therefore, seems most appropriate to use, although overlap could result in false-positive and false-negative results. The diagnostic accuracy for post-treatment lymph nodes using the IVIM or DKI methods might be better using multiple *b* values. This remains speculative currently as diagnostic accuracy studies are lacking post-therapy. The values of the known decrease of kurtosis of lymph nodes during treatment [57, 58] should be further established. The IVIM-derived D values represent pure diffusion without perfusion components. Significantly higher D values are demonstrated in patients with regional failure in line with the ADC results [29•, 33•]. However, another study showed no significant rise in *D* values but a higher initial *f* value (perfusion fraction) in locoregional failure compared to locoregional control [53].

### Perfusion-Weighted Imaging

**Fig. 5:**
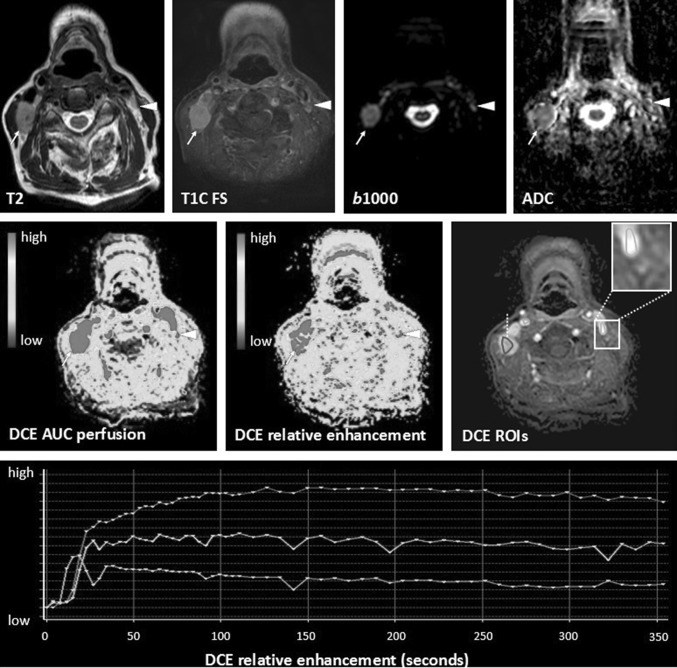
Nodal metastasis with positive diffusion and perfusion. Same patient as in Fig. 4 showing a lymph node metastasis with necrotic center with high T2 signal and no enhancement or increased perfusion (arrow head). Peripheral enhancement corresponded with high AUC (arrows) (Color figure online)

**Fig. 6:**
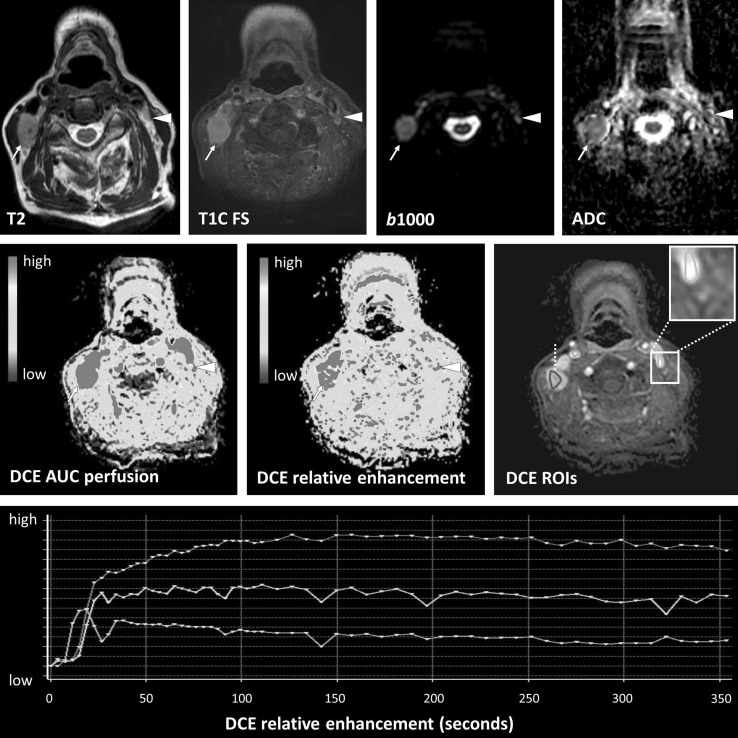
Normal lymph node and nodal metastasis with diffusion and perfusion. A 66-year-old patient with a right sided pT1N1Mx floor of the mouth SCC demonstrated recurrent lymph nodes after postoperative radiation therapy. An enlarged metastasis lymph node was seen on the right side with diffusion restriction and increased relative enhancement and AUC (arrow). A contralateral lymph node was not enlarged and demonstrated slightly restricted diffusion as is also seen in normal lymphoid tissue. Perfusion showed a high AUC and relative enhancement with a rapid wash-in with plateau phase for both lymph nodes, although most pronounced in the metastatic lymph node. Interpretation of the perfusion of lymph nodes remains difficult and should be further investigated (Color figure online)

A few recent studies have demonstrated differences in perfusion parameters between benign lymph nodes and malignant lymph nodes [24••, 33•, 41, 42]. Perfusion of nodal metastasis might be increased (Fig. 5). Metastatic lymph nodes demonstrate higher blood flow and blood volume compared to benign lymph nodes on CT perfusion [41, 42], which thus would be expected to be similar for DSC MRI perfusion. The capillary permeability (Ktrans) correlates with the hypoxia-induced transcription factor in the tissue, which is known to stimulate angiogenesis [59]. However, interpretation of MR perfusion in post-therapy lymph nodes is difficult and it remains to be elucidated whether differentiation of malignant and benign lymph nodes can be done reliable (Fig. 6).

### MR Spectroscopy

Acquiring MRS in lymph nodes is currently not clinically applicable as the region of interest should be placed separately on each suspicious lymph node by a radiologist on site. If the technically challenges are overcome, the increased choline, decreased creatine and subsequently increased choline/creatine ratio of metastatic nodes need to be confirmed in larger studies [60–62].

## Limitations

The limitations and potential pitfalls of the functional MRI sequences should be kept in mind during the interpretation. First, the lack of anatomical information at high *b* values in DWI is a drawback because of suppressed signal in most of the normal tissues. Therefore, DWI should not be interpreted alone, but in correlation with anatomical sequences. This is also true for perfusion and spectroscopy which means that all functional MRI sequences can never be used without the use of anatomical sequences. Moreover, all functional sequences are currently hindered by high variability of cut-offs and parameters used.

Second, it must be stressed that functional MRI remains technically challenging to perform due to artifacts (i.e. breathing, swallowing, involuntary motion and air-tissue interfaces) [10••, 11, 12, 13•, 14•, 24••, 25••, 29•, 32, 33•]. Moreover, acquisition parameters have yet to be standardized. Examples of protocols for the functional MRI sequences of the head and neck are described and could be used as a guide when implementing these sequences [24••].

Diffusion-derived interpretation is mainly done using mean ADC values. Diffusion showed good reproducibility for baseline scans for the ADC value of the primary tumor and nodal metastasis [63]. The reproducibility of the ADC during treatment is also suggested to be good [64]. Mean values of the tumor or metastatic lymph node are not representative when they consist of both highly and poorly cellular (necrotic) portions. Mean ADC values should be measured in the areas with high cellularity only to overcome this limitation [24••, 25••]. Even then, ADC interpretation remains challenging. A recent study suggested a reduced field of view (FOV) might increase accuracy [65]. Moreover, it has been suggested that multiple *b* values are more accurate as this method is able to distinguish the perfusion component resulting in a pure diffusion value. This perfusion might influence the ADC value, although some consider the influence of perfusion below clinical relevance [65]. As the clinical implication of multiple *b* values is not yet firmly established, the acquisition of multiple *b* values in clinical setting can be questioned. However, multiple *b* values are clearly preferred in a research setting.

With respect to DCE perfusion, an increased scan duration with approximately 7–10 min is most hindering clinical applicability next to the potential artifact as discussed above [24••]. DCE perfusion is least influenced by artifacts and currently best suited to perform in patients with HNSCC. Post-processing of perfusion is more complex due to the nonspecific nature of vessel leakage resulting in possible false-negatives and false-positive results. Perfusion post-processing also has a greater range of methods and functional parameters for analysis that are available if compared to DWI [24••, 25••, 41]. This adds to the complexity of perfusion imaging and its clinical implementation.

Studies with regards to MRS suggest a higher choline-to-creatine ratio in patients with poor prognosis, which corresponds with expected high rates of proliferation and membrane biosynthesis in aggressive tumors (increased rate of metabolism) [48]. However, MRS is not commonly used due to its technical challenges. The region of interest should be placed by a radiologist to ensure correct placement in the anatomically difficult head and neck area. Furthermore, motion artifact from the carotid artery, long scan durations and complex post-processing hinders clinical applicability [52, 66].

## Future Developments and Challenges

Differentiation between malignancy and benign post-treatment effects such as fibrosis in HNSCC is of importance to guide clinical decisions. The head and neck is an area sensitive for artifacts and functional MR imaging requires advanced MRI post-processing software to evaluate HNSCC. Combined functional sequences are required to fully appreciate HNSCC post-therapy, in addition to the necessary anatomical sequences. This would result in long scan durations, but new developments could overcome time issues. A possible role of hybrid integrated PET/MR imaging might be demonstrated offering the potential to acquired anatomical and function data using different modalities. However, future research is needed to evaluate PET/MRI and its appropriate applications compared to existing techniques [67] and whether PET/MRI is of greater clinical value than PET/CT and retrospective image fusion techniques [68]. HNSCC is common and local residual and/or recurrence and nodal metastasis are seen in many patients. Diffusion is already frequently used. However, diffusion with multiple *b* values and perfusion required further confirmation of their added value in the post-therapy setting before wide-spread implementation. This is even more the case for spectroscopy. Future studies should focus on the added value of the different functional MRI sequences preferable by large prospective longitudinal multicenter studies comparing all sequences in the same population.

These studies are needed to assess the diagnostic accuracy of the functional MRI sequences separately and in combination. Another important aspect of these studies should be to define the optimum time for assessment of metabolic and physiological MRI parameters using functional techniques. The functional parameters should be tested in relation to the histopathological changes in HNSCC, treatment effects and patient outcomes. These new trials must result in standardized cut-off values and ratios for the anatomical and functional MRI sequences to precisely define post-therapy changes from tumor progression. The use of standardized cut-off values might remain arbitrary because of the use of different MRI systems. Nevertheless, it would be a valuable guideline for the clinician in daily practice. Despite these possible limitations, implications into clinical practice would be an important step in making an accurate treatment decisions for HNSCC patients.

## Conclusions

In summary, this review analyzed the role of specific functional MRI modalities in differentiating benign post-treatment effects from recurrence and/or residual malignancy and metastases in HNSCC.

Differentiation between malignant and benign post-treatment effects in HNSCC is of importance to guide clinical decisions. As anatomical MRI is not able to reliably differentiate post-therapy effect from tumor, functional techniques have been investigated and shown to be promising. This review showed that DWI can increase the diagnostic accuracy significantly for the primary tumor site and might also increase the diagnostic accuracy for the region lymph nodes after therapy. Diffusion is most easy to implement and is recommended to perform routinely in a clinical setting in HNSCC follow-up. Its use during treatment to predict outcome is interesting, but evidence is too low to implement.

Although perfusion parameters might be increased in tumor residual or recurrence and nodal metastasis, its diagnostic accuracy has yet to be established and is not routinely used clinically. DCE is least hindered by artifact and might be performed clinically if local experience is present.

Spectroscopy research is promising, but evidence is too sparse for clinical implementation in the near future. The role of hybrid PET/MR imaging is to be established.

## Abbreviations

ADC: Apparent diffusion coefficient
ASL: Arterial spin labeling
DCE: Dynamic contrast enhanced
DKI: Diffusion kurtosis imaging
DSE: Dynamic susceptibility enhanced
DWI: Diffusion weighted imaging
HNSCC: Head and neck squamous cell carcinoma
IVIM: Intravoxel incoherent motion
